# Association between Psychosocial Working Conditions and Perceived Physical Exertion among Eldercare Workers: A Cross-Sectional Multilevel Analysis of Nursing Homes, Wards and Workers

**DOI:** 10.3390/ijerph16193610

**Published:** 2019-09-26

**Authors:** Leticia Bergamin Januario, Kristina Karstad, Reiner Rugulies, Gunnar Bergström, Andreas Holtermann, David M. Hallman

**Affiliations:** 1 Department of Occupational Health Sciences and Psychology, Centre for Musculoskeletal Research, University of Gävle, Kungsbäcksvägen 47, 801 76 Gävle, Sweden; Gunnar.Bergstrom@hig.se (G.B.); David.Hallman@hig.se (D.M.H.); 2 National Research Centre for the Working Environment, Lersø Parkallé 105, 2100 Copenhagen, Denmark; kka@nfa.dk (K.K.); rer@nfa.dk (R.R.); aho@nfa.dk (A.H.); 3 Department of Public Health, University of Copenhagen, Øster Farimagsgade 5, 1353 Copenhagen, Denmark; 4 Department of Psychology, University of Copenhagen, Øster Farimagsgade 2A, 1353 Copenhagen, Denmark; 5 Unit of Intervention and Implementation Research for Worker Health, Institute of Environmental Medicine, Karolinska Institutet, Nobels Väg 13, 171 65 Stockholm, Sweden

**Keywords:** healthcare, multilevel analysis, nursing home, physical exertion, psychosocial factors, workload

## Abstract

This cross-sectional multilevel study aims at investigating the associations between psychosocial working conditions of different workplace levels and perceived physical exertion among eldercare workers. Data were obtained from the ‘Danish Observational Study of Eldercare work and musculoskeletal disorderS’ (DOSES) study, including 536 eldercare workers, nested in 126 wards and 20 nursing homes. Psychosocial working conditions were measured by the Copenhagen Psychosocial Questionnaire (COPSOQ). The physical workload was measured with a self-administered scale (0–10) rating perceived physical exertion. Multilevel linear mixed models were used to determine associations of psychosocial conditions between nursing homes, wards, and workers with physical exertion. Most of the variance in the perceived physical exertion was explained by differences between workers (83%), but some variance was explained by wards (11%) and nursing homes (6%). Workers employed in nursing homes with low influence (*p* = 0.01) and poor leadership (*p* = 0.02), and in wards with high quantitative demands (*p* = 0.03), high work pace (*p* < 0.001), and low justice (*p* = 0.01) were at increased risk of reporting higher physical exertion. The strongest associations were found for low influence, low quality of leadership, and high work pace at nursing homes and ward levels. In conclusion, improving specific psychosocial working conditions at nursing home and ward levels may be of particular importance to reduce excessive physical workload in eldercare workers.

## 1. Introduction

Eldercare workers are exposed to high physical workloads, such as heavy lifting, resident handlings and awkward body postures, which may explain the high prevalence of musculoskeletal symptoms [1,2], reduced work ability [3], and sickness absence [4]. High perceived physical exertion at work, a common measure of overall physical workload, is a strong predictor of musculoskeletal pain [5,6], higher risk for disability pension [7,8] and early retirement intentions [9] among eldercare workers.

Despite efforts to improve ergonomics and prevent injuries for eldercare workers, for example, by implementing assistive devices [10,11], interventions have been proven only partially effective [10,12,13]. One reason may be that interventions have not targeted organizational and psychosocial working conditions [14,15], which likely have an impact on biomechanical exposure and physical workload in healthcare settings [16]. For example, high levels of control and influence may allow the worker to plan the execution of physically demanding tasks, such as resident handling [2,17], while insufficient influence, low social support, and lack of communication may hinder the use of assistive devices and asking colleagues for help if needed [18]. Good leadership and high organizational justice could create better planning and fair allocation of work tasks between workers, which may reduce high physical workloads [19,20,21,22]. In contrast, high job demands (e.g., quantitative demands, work pace, and emotional demands) may lead to work intensification and more frequent occurrence of heavy lifting and awkward body postures throughout the working day [17,23,24,25]. 

Even though some previous studies suggest that psychosocial working conditions may affect the physical workload among eldercare workers [2,18], further knowledge is important for developing guidelines and effective interventions to prevent the development of health problems in eldercare workers [15,24,26].

Understanding the relationship between psychosocial working conditions and physical exertion requires adopting a multilevel approach that addresses such potential relationships at different workplace levels, for example, between nursing homes, wards, and workers. This is important for interventions in order to target specific workplace factors at the appropriate levels (e.g., focusing on individual workers within a specific ward or on the whole ward or nursing home) [27,28]. In this study, we use aggregated data from the workers into higher workplace levels [29,30], which provides estimates for each level independent of the others and reduces the risk of bias due to individual factors [31]. In addition, we used data collected from the team managers and upper managers, as this could yield better estimates concerning the higher levels [28]; for example, communication among the managers and how work tasks are distributed across workers within a team [22,32].

The aim of this multilevel study is to investigate the extent to which psychosocial working conditions are associated with the physical exertion of eldercare workers. Using data provided by the workers, we investigated the associations between psychosocial working conditions (at the (i) nursing home, (ii) ward, and (iii) individual level) and workers’ perceived physical exertion, and evaluated the amount of variance explained by each of the three levels. We also investigated the associations between psychosocial working conditions reported by the managers responsible for the nursing homes (upper managers) and the wards (team managers) and workers’ perceived physical exertion.

## 2. Materials and Methods 

### 2.1. Study Population

We used data from the ‘Danish Observational Study of Eldercare work and musculoskeletal disorderS’ (DOSES). The study design and protocol has been described previously [33]. Briefly, data were collected at nursing homes located in the Copenhagen area, and 83 nursing homes were invited to participate. After initial contact and participation agreement, 20 nursing homes with an average of 6.3 (SD 3.1) wards, 79 (SD 28.9) residents, and a total of 941 eldercare workers eligible for participation, were included in the study.

The following criteria were used to assess eligibility for the eldercare workers: age between 18 and 65 years old, employed more than 15 h per week, work on days, evenings, or changing shifts, and with at least one-quarter of their working time on tasks related to direct care of residents. From the 941 eligible eldercare workers, 815 responded to the screening questionnaire, and 624 answered that they wished to participate in the DOSES study. The interested eldercare workers were invited to a 45 min session with the administration of a questionnaire, measurements of health and capacity outcomes, and further instructions about the DOSES data collection. From the 624 interested eldercare workers, 71 were excluded, due to nonattendance to the session or failure to return the baseline questionnaire.

**Figure 1:**
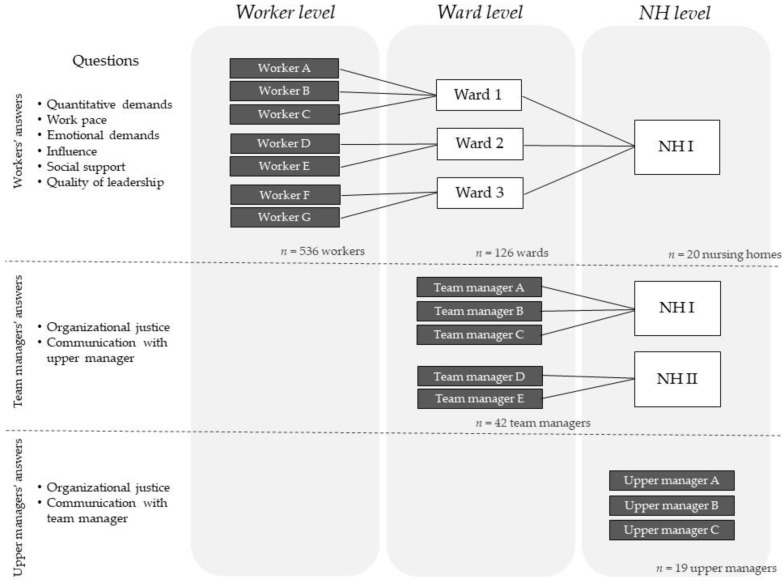
Schematic draft of multi-level data structure with worker, ward, and nursing home (NH) levels. The black boxes represents the data origin, retrieved from the answers obtained from the questionnaires. The questions were specific for each level and were mentioned in the figure. The answers were aggregated into higher levels and are represented by the white boxes.

Initially, 553 eldercare workers distributed into 126 wards and 20 nursing homes were included in the cross-sectional analyses of DOSES. The wards were supervised by team managers (*n* = 42), and the nursing homes were supervised by upper managers (*n* = 19). From the initial sample, 17 eldercare workers were excluded due to missing information about their managers, resulting in a total of 536 eldercare workers, as schematized in Figure 1. The study was approved by the Danish Data Protection Agency and the Regional Ethics Committee in Copenhagen, Denmark (H-4-2013-028). All participants provided their written, informed consent to participate.

### 2.2. Data Collection

We obtained data from self-administrated questionnaires, provided to eldercare workers, team managers, and upper managers during baseline, collected from September 2013 to December 2014. DOSES follows a hierarchical structure with three workplace levels. The eldercare workers were considered as the first level (worker level), the team managers supervising the wards as the second level (ward level), and the upper managers supervising the nursing homes as the third and highest level (nursing home level). Figure 1 shows the different levels of the multilevel data structure with the origin of the data and the aggregation steps across levels.

#### 2.2.1. Psychosocial Working Conditions (Predictors)

We measured different psychosocial working conditions using the Copenhagen Psychosocial Questionnaire (COPSOQ) [34] that were treated as predictor variables. 

The eldercare worker questionnaire contained six COPSOQ scales [34] assessing: quantitative demands (two items—QD3, QD4), work pace (two items—WP1, WP2), emotional demands (four items—ED1, ED2, ED3, ED4), influence (two items—IN1, IN3), social support (three items—SC1, SC2, SC3), and quality of leadership (four items—QL1, QL2, QL3, QL4). All questions used a five-point Likert scale from 0 (‘always/to a very large extent’) to 5 (‘never/to a very small extent’). The answers were converted to a score of 0–100 before averaging the items for each scale, whereby higher scores express higher levels of the measured psychosocial working condition [34]. The answers obtained from the eldercare worker questionnaires were aggregated to ward and nursing home levels; therefore, the responses to these variables were available at all three levels (Figure 1). 

The team manager questionnaire included one question about communication with the upper manager using a numeric rating scale from 0 (‘very little’) to 10 (‘very much’); and one question about organizational justice (“To what extent do you think that the tasks are distributed in a fair way between your employees?”) based on the COPSOQ question (JU4) [34]. As the team managers often supervised more than one ward at the time, they should provide one answer about the organizational justice per ward supervised. The answers obtained from the team manager questionnaires were also aggregated to nursing home level; therefore, the responses to these variables were available at both ward and nursing home levels (Figure 1). 

The upper manager questionnaire included one question about sufficient communication with team managers using a numeric rating scale from 0 (‘very little’) to 10 (‘very much’) and the same question about organizational justice based on the COPSOQ question (JU4) [34]. 

#### 2.2.2. Perceived Physical Exertion (Outcome)

Perceived physical exertion was used as the outcome and assessed from the workers using the question: “How physically demanding do you normally consider your present work?” using an 11-point numeric rating scale [35] from 0 (‘not demanding’) to 10 (‘extremely demanding’). The perceived physical exertion is a general measure of overall physical workload and reflects the balance between the physical work demands and the physical capacity of the worker [8,36,37]. 

#### 2.2.3. Covariates 

Various covariates were selected a priori based on theory and empirical studies of their relationship with psychosocial working conditions and perceived physical exertion. Age (years), gender (man or woman), education (social and health service aides, social and health service helpers, or other types of education) [38], shift (day, evening, or changing shifts) [39], and leisure-time physical activity [40] were assessed based on the worker’s baseline questionnaire. Leisure- physical activity was evaluated based on the questions proposed by Saltin and Grimby [40] and classified according to Andersen et al. [41] into low (mainly sedentary or light physical activity <2 h/week), medium (light physical activity 2–4 h/week), high (light physical activity >4 h/week or vigorous physical exercise 2–4 h/week), and very high (vigorous physical exercise >4 h/week or taking part in regular competitive sports several times a week). The body mass index (BMI = kg/m^2^) was also considered as a covariate and calculated based on objective measures of height and weight obtained during an individual 45 min session with the administration of the questionnaires and measurement of health and physical capacity [33]. Based on the data provided by the team managers, we assessed the type of ward [21] (three categories: somatic unit, dementia or other) and staffing ratio [42] (the number of eldercare workers in a determined shift divided by the number of residents in each ward).

### 2.3. Statistical Analysis

Since DOSES data follow a hierarchical structure, we adopted a multilevel linear mixed model approach for the statistical analyses [43], considering ward and nursing home as random effects. We used the Statistical Package for the Social Sciences (SPSS, v 24.0, IBM, Armonk, NY, USA) for all analyses. Effect estimates were expressed as beta (β) with 95% confidence intervals (CI), and *p*-values < 0.05 indicated statistical significance. 

Three sets of multilevel models were run to determine (i) the proportion of explained variance in both predictors (psychosocial working conditions) and the outcome (perceived physical exertion); (ii) the crude association between each psychosocial working condition and perceived physical exertion; and (iii) the adjusted associations between psychosocial working conditions and perceived physical exertion. 

First, we obtained variance components across the three levels for physical exertion using a null model, based on a random effect model with no predictors. The intraclass correlation coefficient (ICC) was calculated to determine the proportion of variance in perceived physical exertion explained by each of the three levels. We used the same models to determine the ICCs for each psychosocial working condition [44]. 

Second, we determined the crude association of psychosocial working conditions with perceived physical exertion by adding the psychosocial working conditions as predictors to the null model (explained above). For each psychosocial condition (entered in separate models), we determined the independent effect of each level by entering the worker data, the mean across workers within wards, and the mean across wards within nursing homes as predictors in the same model. Thus, the effect of each level was adjusted by the other levels. Prior to the analysis, we mean-centered all predictor variables to avoid possible multicollinearity [45]. 

Third, we run all models with additional adjustment for the covariates age, gender, education, BMI, leisure-time physical activity, shift, type of ward, and staffing ratio. 

The three sets of multilevel models explained above were also constructed to determine associations of psychosocial working conditions, based on data provided by the team managers and upper managers, with workers ratings of physical exertion. Considering that both predictors and outcome variables were based on self-reported data, we accounted for possible common method variance using Harman’s single factor test [46]. As the total percentage of variance for all the modeled self-reported data was just 26%, i.e., below 30% [46], the common method variance was not considered a major concern in this study. 

## 3. Results

### 3.1. Descriptive Characteristics of the Study Population

The eldercare workers were on average (SD), 45.6 (10.9) years old and had a BMI of 26.5 (5.3) kg/m^2^. Most of them were women (95%) and worked in day shifts (55%), 25.4% worked in evening shifts, and 19.6% in changing shifts. Regarding the education, 37.3% were social and health service aides, 46.6% social and health service helpers, and 15.6% had other education. The staffing ratio at wards was on average 0.47 (0.12) worker per resident, 0.32 (0.07) in day shift, and 0.15 (0.06) in the evening shift. Regarding the type of wards, 75.2% were somatic units, 20.3% were dementia units, and the remaining 4.5% of the wards were from other units. 

Table 1 shows descriptive data and variance components across nursing homes, wards, and workers for physical exertion (outcome) and psychosocial working conditions (predictors). The mean physical exertion was 6.8 (SD 2.0) (scale 0–10), with most variance explained by differences between workers (83%) and less variance explained by wards (11%) and nursing homes (6%). For psychosocial data provided by workers, more than 85% of the variance in all psychosocial working conditions was explained by differences between workers. The only exception was the quality of leadership showing a considerable amount of variance explained by wards (13%) and nursing homes (19%). For psychosocial data provided by team managers, a larger proportion of the variance in communication was explained by differences between nursing homes than wards (Table 1). 

### 3.2. Association between Psychosocial Conditions Reported by Workers and Physical Exertion

Table 2 shows the association between psychosocial working conditions based on data provided by the workers and physical exertion, with estimates for all three levels. At nursing home level (effect between nursing homes), strong influence and high quality of leadership were significantly associated with lower perceived physical exertion after adjustments. At ward level (effect between wards within nursing homes), high quantitative demands, and high work pace were significantly associated with higher perceived physical exertion in the adjusted model. The other psychosocial working conditions at the ward level were not significantly associated with physical exertion. 

At worker level (effect between workers within wards), all psychosocial working conditions were significantly associated with physical exertion in the adjusted models. Quantitative demands, work pace, and emotional demands were associated with higher levels of physical exertion, while influence, social support, and quality of leadership were associated with lower levels of physical exertion.

### 3.3. Association between Psychosocial Conditions Reported by Managers and Physical Exertion

Table 3 shows the association between psychosocial working conditions, based on data provided by the team and upper managers, and physical exertion reported by eldercare workers. The team managers’ perceptions of high organizational justice were significantly associated with lower perceived physical exertion at ward level, but not at the nursing home level. The association of communication with physical exertion was not statistically significant at any level, and no effect was found based on data provided by the upper managers.

## 4. Discussion

This study used a multilevel approach to investigate the association between psychosocial working conditions and perceived exertion in eldercare workers, comparing nursing homes, wards, and individual workers. Overall, we found that perceived physical exertion was associated with various psychosocial working conditions across the three workplace levels. We found larger associations for the higher levels (i.e., nursing home and ward), which may be particularly important regarding the targeted prevention of excessive workload and its negative consequences in eldercare work.

### 4.1. Psychosocial Data Provided by Eldercare Workers

The main finding of this study is the observed associations for psychosocial working conditions at the higher levels (ward and nursing home) with physical exertion, regardless of the worker level psychosocial working conditions and potential confounders. Specifically, we found that in nursing homes characterized by high levels of influence (β = −0.066) and quality of leadership (β = −0.047), eldercare workers reported lower levels of physical exertion compared to nursing homes characterized by low levels of influence and quality of leadership. In practical terms, a ten points increase in the influence and quality of leadership in nursing homes (0–100 scale), was associated with 0.66 and 0.44 units decrease in physical exertion reported by the eldercare workers (0–10 scale). This could be of relevance for reducing risks of musculoskeletal disorders [5,6] and sickness absence [37].

One possible explanation for this finding is that influence (e.g. the possibility to make decisions concerning work) and quality of leadership (e.g., managers prioritizing work satisfaction, planning the workday, and solving conflicts) may reduce work stress, which may increase the ability to cope with poor physical working conditions [21,47]. Also, the workers may have better possibilities to plan their work, which may help to reduce physically heavy or uncomfortable work tasks [2]. These findings suggest that promoting leadership and influence at the overall nursing home level may be more effective for avoiding excessive physical workload among the eldercare workers compared to targeting wards or individual workers. 

At the ward level, work pace (β = 0.045) and quantitative demands (β = 0.025) showed positive associations with physical exertion in the adjusted models, while influence, social support, and the quality of leadership were not statistically significant. This indicates that, within a nursing home, wards with higher job demands are associated with higher physical exertion among the workers. This finding may have implications for interventions targeting specific wards to reduce excessive physical workload. Although we adjusted for type of ward, it is possible that wards have different job demands depending on the type of residents. For example, in wards where the residents are more dependent, the eldercare workers face higher job demands which can partially explain the current results [48,49]. However, the specific modifying factors contributing to the associations between psychosocial working conditions and physical exertion and the effects of the selected covariates at the different levels are still unknown and future research is needed.

We found that nursing homes and wards explained significant proportions of variance in quantitative demands, work pace and quality of leadership (Table 1), which is consistent with a previous study in Danish eldercare workers [29], although we found a larger contribution of the quality of leadership for the higher workplace levels. The combined variance in influence explained by nursing homes and wards was nearly 10%, but the independent contributions were not statistically significant. 

We found that high job demands and low job resources were both associated with higher physical exertion at the worker level, and those associations were slightly larger for work demands. These results corroborate previous studies [37,39] and suggest that, within wards, elderly care workers reporting lower job demands (i.e., quantitative demands and work pace) and higher job resources (i.e., quality of leadership, influence and social support) perceive lower physical exertion. Positive associations of quantitative demands and work pace with perceived physical exertion were expected, as high job demands may lead to direct changes in the amount and intensity of physical work [50], which could increase biomechanical exposure [51,52] and result in higher perceived physical exertion [51]. Our results did not change after adjustment for age, gender, education, BMI, leisure-time physical activity, shift, type of ward, and staffing ratio. Still, it is important to interpret these findings with caution as both exposure and outcome were reported by the workers and were analyzed on the individual level.

### 4.2. Psychosocial Data Provided by Managers

In wards where the team managers perceived the tasks as distributed fairly between workers (organizational justice), the eldercare workers perceived lower physical exertion. This finding may be explained by the fact that team managers may distribute the tasks equally among the eldercare workers according to their physical capacity in certain wards, which may lead to an evenly distributed physical workload that reflects on the perceived physical exertion. Previous studies have shown that organizational justice is positively associated with general worker health and ability to cope with uncertainty and stress in the work environment [53]. Based on the psychosocial data provided by upper managers, no statistically significant association with the perceived physical exertion of eldercare workers was found. 

A general recommendation for interventions in workplaces with high physical workload would be to focus simultaneously on physical aspects (e.g., including assistive devices and ergonomic guidelines about working postures) and targeting psychosocial working conditions in different levels to improve the effectiveness and the quality of interventions [54,55]. The effect of different levels can be used to understand how interventions should target workers within wards, specific wards within nursing homes, or specific nursing homes. 

### 4.3. Methodological Considerations

A large number of workers and managers allowed us to investigate the associations at different levels, while also generalizing the findings to a broader population. However, it is important to consider that higher hierarchical structures, i.e., ward and nursing homes, have fewer units when compared with the workers’ units, which affect the statistical power of the analyses.

The main strength of this study is the multilevel approach, which is important to understand what specific psychosocial factors should be targeted at different workplace levels to reduce physical workload among the eldercare workers and if interventions should target the whole organization, specific wards or individuals [27,28]. To our knowledge, this is the first study to consider three levels in a multilevel analysis of the effects of psychosocial working conditions on perceived physical exertion in eldercare workers. As workplace characteristics can be quite different between wards, even within the same nursing home, we analyzed data for higher workplace levels separately for nursing homes and wards. In contrast, other multilevel studies have mainly considered one higher level [29,30,56]. Another strength is that we collected psychosocial data both from workers and managers, considering independent data in each workplace level [28]. 

The study also has some limitations. First, our data are based on a cross-sectional design and causal relations between physical and psychosocial working conditions cannot be addressed. Even though the cross-sectional design is not a major concern when evaluating the aggregated data to ward and nursing home levels or when evaluating the data obtained directly from the team managers and the upper managers, the cross-sectional design is a concern when analyzing data obtained at the worker level. Besides, the questionnaires were different for managers and workers, and some psychosocial working conditions, such as organizational justice and communication, were rated exclusively by the managers, which may not be consistent with the perception of such conditions among the eldercare workers.

The use of self-reported measures of both exposures and outcome may result in common method variance, which could be a potential source of biased relationships, at least in the workers level results. Still, we addressed the common method variance using Harman’s single-factor test, a post hoc procedure based on principal components analysis, used to address the issue of common method variance [46]. We found that the percentage of variance for all modeled self-reported variables in this study corresponded to 26%, indicating that common method variance was not a serious problem in this data [46,57]. However, this possible issue should be considered when interpreting the results. 

In general, this study of Danish eldercare workers reported slightly higher job demands and higher perceived physical exertion when compared with previous studies, but also reported better job resources (i.e., influence, social support and quality of leadership) [37,39,58]. It is possible that the relationship between psychosocial working conditions and physical exertion will differ in other populations.

Also, the variance explained by ward and nursing home levels was relatively small for some psychosocial conditions, particularly for emotional demands and social support. This may have resulted in attenuated estimates of associations with physical exertion, which therefore requires cautious interpretation [29,30].

Even though perceived physical exertion is a good indicator of relative physical workload, i.e., reflecting the balance between the physical work demands and the physical capacity of the worker [35,37], objective measurements would likely have provided more accurate information about the absolute physical workload [59,60,61]. Future studies should consider investigating objective measures of working conditions, such as direct observations or technical measurements of working postures and conditions of resident handling, in order to bring new insights on the association between psychosocial and physical working conditions and in relation to occupational health.

## 5. Conclusions

We found that various psychosocial working conditions at different workplace levels were associated with perceived physical exertion among eldercare workers, and that psychosocial job resources at higher levels were of particular importance in this respect. For example, in nursing homes with stronger influence and better leadership among the workers, and in wards with team managers reporting fairer task distribution, the workers reported significantly lower perceived physical exertion. Overall, our findings provide new insights into the interplay between psychosocial working conditions at different workplace levels and physical workload, which can aid preventive efforts and interventions to reduce detrimental physical workload among eldercare workers.

## Figures and Tables

**Table 1:** Mean and standard deviation (SD) between workers (*n* = 536), and variance with 95% confidence interval (CI) between nursing homes, wards, and workers for physical exertion and psychosocial working conditions. The intraclass correlation coefficient (ICC) is expressed as the percentage of explained variance by each level.

Variables	Mean (SD)	Variance Components								
		Nursing Home Level			Ward Level			Worker Level		
		Variance	95% CI	ICC	Variance	95% CI	ICC	Variance	95% CI	ICC
Eldercare workers’ responses										
Physical exertion (0–10)	6.8 (2.0)	0.2	0.1–0.7	5.6	0.4	0.2–0.9	11.1 *	3.2	2.8–3.7	83.3 *
Quantitative demands (0–100)	44.4 (20.2)	38.0	14.2–101.4	9.1 *	11.5	1.1–124.3	2.8	365.4	319.5–417.9	88.1 *
Work pace (0–100)	60.9 (18.0)	22.1	6.4–75.8	6.7	22.4	7.7–65.3	6.8 **	283.2	247.7–323.7	86.4 *
Emotional demands (0–100)	51.4 (15.5)	2.9	0.2–35.4	1.2	6.5	0.4–97.7	2.7	232.6	203.0–266.5	96.1 *
Influence (0–100)	57.1 (19.7)	19.0	5.6–64.6	4.9	16.3	2.4–111.5	4.2	353.1	308.1–404.6	90.9 *
Social support (0–100)	71.7 (17.4)	6.3	0.9–43.0	2.5	13.9	3.5–54.9	5.5	232.2	203.2–265.2	92.0 *
Quality of leadership (0–100)	60.6 (17.4)	58.1	25.5–132.2	19.1 *	40.2	21.0–76.9	13.2 *	205.9	179.6–235.9	67.7 *
Team managers’ responses										
Communication (0–10)	8.3 (2.3)	4.8	2.5–9.1	80.5 *	1.2	1.0–1.3	19.5 *			
Justice (0–100)	71.6 (15.5)	117.2	59.3–231.8	44.7 *	145.3	128.6–164.2	55.3 *			
Upper managers’ responses										
Communication (0–10)	9.1 (1.0)	1.0	0.9–1.1	100.0 ^†^						
Justice (0–100)	75.0 (10.8)	116.7	101.1–134.6	100.0 ^†^						

* *p* < 0.05; ** *p* < 0.10; ^†^ no data were obtained at the lower levels.

**Table 2:** Association between workers perceptions of psychosocial working conditions and physical exertion (scale 0–10). Multilevel analysis of effects attributed to nursing homes (*n* = 20), wards (*n* = 126), and workers (*n* = 536). All predictors were based on a 0–100 scale.

Predictors	Crude Model			Adjusted Model ^a^		
	Β	*p*	95% CI	Β	*p*	95% CI
*Quantitative demands*						
Nursing home level	−0.015	0.50	−0.059–0.029	−0.019	0.40	−0.065–0.026
Ward level	0.020	0.07	−0.002–0.041	**0.025**	**0.03**	**0.003–0.048**
Worker level	**0.025**	**<0.001**	**0.016–0.033**	**0.025**	**<0.001**	**0.015–0.034**
*Pace*						
Nursing home level	−0.014	0.55	−0.060–0.032	−0.027	0.24	−0.073–0.019
Ward level	**0.041**	**<0.001**	**0.019–0.062**	**0.045**	**<0.001**	**0.023–0.067**
Worker level	**0.027**	**<0.001**	**0.017–0.037**	**0.026**	**<0.001**	**0.016–0.037**
*Emotional demands*						
Nursing home level	−0.061	0.23	−0.164–0.041	−0.060	0.23	−0.157–0.040
Ward level	0.009	0.51	−0.018–0.037	0.022	0.15	−0.008–0.052
Worker level	**0.019**	**<0.001**	**0.008–0.030**	**0.017**	**0.01**	**0.006–0.029**
*Influence*						
Nursing home level	−0.045	0.07	−0.094–0.004	**−0.066**	**0.01**	**−(0.115–0.016)**
Ward level	0.002	0.86	−0.020–0.024	0.006	0.58	−0.017–0.030
Worker level	**−0.017**	**<0.001**	**−(0.027–0.008)**	**−0.019**	**<0.001**	**−(0.029–0.010)**
*Social support*						
Nursing home level	−0.008	0.83	−0.083–0.067	−0.032	0.38	−0.110–0.042
Ward level	−0.018	0.18	−0.045–0.008	−0.009	0.52	−0.039–0.020
Worker level	**−0.012**	**0.04**	**−0.023–0.000**	**−0.013**	**0.04**	**−(0.025–0.001)**
*Quality of leadership*						
Nursing home level	−0.034	0.09	−0.074–0.005	**−0.047**	**0.02**	**−(0.086–0.008)**
Ward level	0.014	0.29	−0.012–0.040	0.016	0.24	−0.011–0.044
Worker level	**−0.017**	**0.01**	**−(0.030–0.005)**	**−0.013**	**0.05**	**−0.025–0.000**

^a^ adjusted for age, gender, education, body mass index, leisure-time physical activity, shift, type of ward, and staffing ratio. Bold values represent statistically significant (*p* < 0.05) associations.

**Table 3:** Association of perceptions from the team and upper managers psychosocial working conditions with workers (*n* = 536) perceptions of physical exertion (scale 0–10). A multilevel analysis of effects attributed to nursing homes (*n* = 20) and wards (*n* = 126).

Predictors	Crude Model			Adjusted Model ^a^		
	β	*p*	95% CI	β	*p*	95% CI
Team managers’ responses						
*Communication (0–10)*						
Nursing home level	0.003	0.98	−0.232–0.237	0.022	0.85	−0.218–0.263
Ward level	−0.007	0.94	−0.193–0.180	−0.016	0.87	−0.220–0.186
*Justice (0–100)*						
Nursing home level	0.009	0.58	−0.024–0.042	0.004	0.76	−0.026–0.035
Ward level	**−0.022**	**0.01**	**−(0.038–0.006)**	**−0.022**	**0.01**	**−(0.038–0.005)**
Upper managers’ responses						
*Communication (0–10)*	−0.097	0.50	−0.403–0.208	−0.062	0.63	−0.318–0.194
*Justice (0–100)*	−0.004	0.79	−0.034–0.026	−0.003	0.77	−0.031–0.023

^a^ adjusted for age, gender, education, body mass index, leisure-time physical activity, shift, type of ward, and staffing ratio. Bold values represent statistically significant (*p* < 0.05) associations.
